# Consumption of energy drinks among Turkish University students and its health hazards

**DOI:** 10.12669/pjms.35.2.638

**Published:** 2019

**Authors:** Arda Borlu, Belgin Oral, Osman Gunay

**Affiliations:** 1 *Arda Borlu, M.D. Assistant Professor Erciyes University Medicine Faculty, Public Health Department, Kayseri, Turkey*; 2 *Belgin Oral, MD HSU Ataturk Chest Diseases and Thoracic Surgery Training and Research Hospital, Ankara, Turkey*; 3 *Prof. Osman Gunay, M.D Erciyes University Medicine Faculty, Public Health Department, Kayseri, Turkey*

**Keywords:** Energy Drink, University student, Alcohol mixed energy drink

## Abstract

**Objective:**

To determine the awareness, consumption patterns of energy drinks (ED) and health hazards among students at a Turkish University.

**Methods:**

This cross-sectional descriptive study was conducted by questionnaire method between in 2017 among Erciyes University students. Total 1257 students from the Faculties of Medicine, Communication and Physical Education and Sports College participated in the study. Pearson chi-square test, binary logistic regressions were used for statistical analysis. p <0.05 values were considered significant.

**Results:**

Students who tried at least once and consumed regularly ED were 52.5% and 15.7% respectively. Consuming regularly and trying ED were more common among students who were studying at Physical Education and Sports High school, male, smoking, alcohol consumer, doing regular physical activity. Mean age of students to start drinking ED was 15.1 years. Most common reason for ED consumption was; staying awake. Alcohol mixed energy drinks consumption rate was 37.6% among regular ED consumers. Most declared harmful effect was palpitation.

**Conclusion:**

ED consumption among Erciyes University students was widespread. Students should be informed about EDs’ hazards for health. Legal regulations regarding production, marketing and advertising of EDs must be reconsidered.

## INTRODUCTION

Although EDs must be consumed carefully due to their contents, young people consume them widely, because of lack of knowledge and misleading advertisements. The most controversial content of ED is caffeine. Irritability, anxiety, restlessness, insomnia, tachycardia are common side effects of caffeine.^1^ Adverse effects are not expected with caffeine intake below 6 mg/kg/day for adults and 2.5 mg/kg for children.^2^ Body weight and personal caffeine sensitivity are predictors for toxic doses.^3^ The amount of caffeine per box of ED ranges from 72 to 294 mg.^4^ Hypertensive individuals are more susceptible to caffeine.^5^ As well as caffeine, ED includes taurine, guarana, ginseng, yohimbine, inositol, B vitamins, and glucuronolactone.^6^ These substances can be stimulant, aphrodisiac, hepatotoxic, nephrotoxic and cardio toxic.^7^

Consumption of AMED (alcohol mixed ED) is often preferred because it causes the person to notice the effects of alcohol later and thus causes more and longer alcohol consumption.^8^ AMED is also common among university students.^9^,^10^ People consuming AMED were shown to be more frequently taking on risky behaviors.^11^

EDs are sometimes consumed instead of sports drinks which are produced to replace water and minerals after sports and do not contain stimulant substances.^12^

Determining the population at risk for ED consumption would be beneficial to develop strategies for reducing the misuses of ED among university students. The aim of this study was to determine the awareness, consumption patterns of ED and related health hazards among students at a Turkish University.

## METHODS

This cross-sectional descriptive study was conducted by questionnaire method between April-June 2017. The universe of study was constituted by students at Erciyes University in 2016-2017 academic year. Sample size was calculated by assuming university students’ EDs consumption rate of 50%. Accepting confidence level 95%, power 90% and tolerance value 0.05, minimum sample size was calculated as 1049. Students studying in first four years of Faculties of Medicine, Communication and Physical Education and Sports College (PESC) were included in the research (3954 students).

Ethical approval from Erciyes University Ethical Committee for Clinical Investigations and administrative permission from schools administrations were obtained for research.

Data were collected by the researchers using a questionnaire consisting of 41 questions. The questions were prepared to determine the participants’ socio-demographic characteristics, awareness and consumption patterns of ED. Students were visited in their classes by researchers, their consent were obtained and filled out the surveys themselves. Students who were not in the class during data collection were not included in this study. Total of 1257 students’ data were evaluated.

Students who reported that they drank ED at least once in their lifetime were accepted as ‘tried ED’ and those who reported that they consume ED at least once a month were accepted as ’regular ED consumer’. Statistical analysis was performed on SPSS 15.0 at Windows program. Pearson’s Chi-square test was used to compare independent variables between trying and regular ED consuming. Binary logistic regression analyses were used for determining related factors for tried ED and regular ED consumers. p<0.05 were considered to be significant.

## RESULTS

Percentages of trying ED and regular ED consumers were 52.5% and 15.7%. Mean initial drinking age of students for ED was 15.1 ± 3.2 years. Factors that have the greatest effect on the first trial were; friends (51.8%) and advertisements (30.9%).

Students’ school, age, gender, doing regular physical activity, smoking and using alcohol situations were found to be related to their both trying and regular ED consuming situations (Table-I). Logistic regression analyses of students’ some characteristics and trying ED were given in Table-II while consuming regularly ED were given in Table-III.

**Table-I:** Trying and Consuming Regular ED According to Characteristics of the Students.

Characteristics	Groups	n	Tried ED		X^2^	p	Consuming regular ED		X^2^	p
	
			Number	%			Number	%		
School	Medicine	632	247	39.1	116.63	<0.001	34	5.4	111.53	<0.001
	Communication	311	182	58.5			67	21.5		
	Physical Education and Sport	314	237	75.5			96	30.6		
School grade	1	346	170	49.1	7.71	0.052	52	15.0	2.78	0.427
	2	335	192	57.3			58	17.3		
	3	304	171	56.3			40	13.2		
	4	272	133	48.9			47	17.3		
Age (year)	19 and under	334	145	43.4	16.72	<0.001	34	10.2	10.38	0.001
	20 and over	923	521	54.6			163	17.7		
Gender	Male	614	394	64.2	60.29	<0.001	118	19.2	11.42	0.001
	Female	643	272	42.3			79	12.3		
Economic situation	High	525	275	52.4	0.22	0.895	86	16.4	2.12	0.347
	Moderate	676	362	53.6			99	14.7		
	Poor	56	29	51.8			12	21.4		
Doing physical egzersize	Never	195	83	42.6	31.61	<0.001	21	10.8	17.69	<0.001
	Irregular	833	424	50.9			120	14.4		
	Regular	229	157	68.9			56	24.5		
Self reported health situation	Good	894	482	53.9	1.83	0.400	144	16.2	2.73	0.256
	Moderate	318	158	49.7			43	13.5		
	Poor	45	24	53.3			10	22.2		
Smoking	Yes	271	196	72.3	51.88	<0.001	80	29.5	50.13	<0.001
	No	986	470	47.7			117	11.9		
Alcohol use	Yes	233	167	71.7	40.11	<0.001	86	36.9	97.61	<0.001
	No	1024	499	48.7			111	10.8		
Having chronic illness	Yes	158	91	57.6	1.54	0.215	30	19.0	1.48	0.224
	No	1099	575	52.3			167	15.2		
Using medicine	Yes	105	62	59.0	1.67	0.197	16	15.2	0.19	0.890
	No	1152	604	52.4			181	15.8		

Total		1257	666	53.0			197	15.7		

**Table-II:** Effect of Characteristics of Students on Trying EDs.

Characteristics	Groups	n	Tried EDs		OR (CI 95%)

			Number	%	
School	Medicine	632	247	39.1	1.00
	Communication	311	182	58.5	2.02 (1.48–2.73)
	Physical Education and Sport	314	237	75.5	3.92 (2.78–5.52)
School grade	1	346	170	49.1	1.00
	2	335	192	57.3	1.31 (0.91–1.88)
	3	304	171	56.3	0.95 (0.62–1.45)
	4	272	133	48.9	0.85 (0.55–1.32)
Age (year)	19 and under	334	145	43.4	1.00
	20 and over	923	521	54.6	1.12 (0.77–1.63)
Gender	Female	643	272	42.3	1.00
	Male	614	394	64.2	2.18 (1.68–2.82)
Doing physical egzersize	Never	195	83	42.6	1.00
	Irregular	833	424	50.9	1.39 (0.98–1.98)
	Regular	229	157	68.9	1.88 (1.19–2.98)
Smoking	No	986	470	47.7	1.00
	Yes	271	196	72.3	1.73 (1.23–2.43)
Alcohol use	No	1024	499	48.7	1.00
	Yes	233	167	71.7	1.61 (1.13–2.30)

Total		1257	666	53.0	

**Table-III:** Effect of Characteristics of Students on Regular Consuming EDs.

Characteristics	Groups	n	Regular EDs Drinkers		OR (CI 95%)

			Number	%	
School	Medicine	632	34	5.4	1.00
	Communication	311	67	21.5	4.15 (2.59–6.66)
	Physical Education and Sport	314	96	30.6	5.43 (3.41–8.65)
School Grade	1	346	52	15.0	1.00
	2	335	58	17.3	1.08 (0.65–1.79)
	3	304	40	13.2	0.70 (0.40–1.24)
	4	272	47	17.3	1.07 (0.61–1.88)
Age (year)	19 and under	334	34	10.2	1.00
	20 and over	923	163	17.7	1.07 (0.62–1.83)
Gender	Female	643	118	19.2	1.00
	Male	614	79	12.3	1.24 (0.87–1.78)
Doing sports	Never	195	21	10.8	1.00
	Irregular	833	120	14.4	1.59 (0.93–2.74)
	Regular	229	56	24.5	2.29 (1.21–4.30)
Smoking	No	986	117	11.9	1.00
	Yes	271	80	29.5	1.52 (1.02–2.26)
Alcohol use	No	1024	111	10.8	1.00
	Yes	233	86	36.9	3.22 (2.18–4.77)

Total		1257	197	15.7	

Consumption characteristics of regular ED drinkers were as followings; most frequent consumption times; during meals (26.9%), before sports (19.3%), after sports (15.2%); most frequent consumption places; café (35.5%), home (33.5%), sports fields (27.9%); common important reasons for consumption; staying awake (15.8%), being strong/fit (11.2%) and habit (8.2%).

AMED consumption rates were 11.1% among whole students and 37.6% among regular ED consumers. Among the regular ED consumers, 14.7 % had a chronic disease, 8.1% were using regular medication. The number of students who said that they had any harmful effect of ED was 6.3%. The most commonly reported hazardous health effect was palpitation (38.1%).

Only 18.0% of the students stated their level of knowledge self-evaluation about as sufficient, 65.6% of them knew ED and sports drinks were not the same. Sugar (82.7%), caffeine (72.9%) and taurine (27.3%) were the most known substances in ED. Most of the students knew that EDs were harmful to pregnant women (81.8%) and children (81.7%). Less than half of the students (40.3%) stated ED as harmful.

## DISCUSSION

Half of the students consumed ED at least once in their lives. This rate was similar to other studies from Turkey; 46.5% among Karadeniz Technical University students^13^ and 48.3% among Hacettepe University students^8^ or from other countries; 54.6% in Puerto Rico^14^ and 51.8% in Taiwan.^15^

The regular ED consumption rate of students was lower than the others researched in the literature (15.7%). This ratio was found to be 33% in a study conducted in Turkey^8^, 51.0% in USA^16^, 24.8% in Taiwan^15^ and 38% in Caribbean.^17^ These differences could be derived both from the various definitions of ’regular ED drinker’ and in terms of having different cultural and economic characteristics of the groups participating in the studies.

The highest percentages of trying and regular consuming of ED were among PESC students. In other studies it was noticed that ED consumption was more common among PESC students than the others.^8^,^13^ This may be due to anticipation of a performance enhancing effect of ED. This idea is also supported by the higher rate of ED consumption among regular physical activity makers than the others in the study. The common misconception that ‘EDs and sports drinks are the same’ may also be increasing the ED consumption rate in physical activity makers. Also in our study, 34.4 % of the participants did not knew ED and sports drinks were different.

Medical students were expected to have more knowledge about EDs’ side effects and to avoid consuming them. As expected, medical students’ drinking ED percentages were lower than others. Bulut et al. reported that medical students were the third among four surveyed faculties according to ED consumption frequency.^13^ At two other studies conducted in only Medicine faculties from Turkey and Pakistan, trying ED percentages was 32.6%^15^ and 42.9% respectively, similar to our study.^18^

Many studies have reported higher trying and regular consuming ED percentages in males than in females, in the literature as in our study.^8^,^13^,^17^,^19^,^20^ Men are more likely to participate in sporting activities and ED consumption is often associated with sporting activities. This may the reason to explain the relationship between men and ED. We noticed friend was the most important factor on first trial of ED drinking as determined in a study from Saudi Arabia.^21^

ED and alcohol consumptions were found to be related in many studies^18^,^20^ as in ours. Another risk about this topic is consumption of AmED. ED cause the effects of alcohol to occur later and by this way leading more alcohol consumption and more exposure to harmful effects of alcohol. In our study, 37.6% of regular ED drinkers consumed AmED. AmED consumption percentages among regular EDs drinkers varies at the researches conducted in different countries; 28.2% in Turkey^13^, 22.2% in Caribbean^17^, 38.0% in Puerto Rico.^14^

It was determined that 6.3% of regular ED drinkers had experienced a harmful effect due to ED, and the most frequent side effect was palpitation. Palpitation was marked as one of the most frequent side effects of ED at many studies.^16^,^17^ Experienced harmful effect rate in our study was lower than experienced at Reid et al. Study (62.2%).^17^ This may be due to our interrogation of side effects with an open-ended question and the inability of students to associate ED with side effects or inability to remember. Common reasons to consume ED were staying awake and being strong/fit. The common reasons determined to consume ED at other studies were; improving physical or academic performance, staying alert.^8^,^16^,^17^

Children are more susceptible to caffeine, ED may be toxic to certain organs, and may interact with certain medicines. For this reason, as stated in the Turkish Food Codex Regulation ED Communique^22^ consumption of ED are not recommended for people under the age of 18, with any chronic disease and drug users. But students’ initial age for ED drinking was 15.1, like in another study from Taiwan (15.7 years).^15^ We also found that 14.7% of the regular ED consumers had chronic disease and 8.1% were using a drug. This results show us that students consume ED unconsciously as they don’t avoid consuming ED even at risky situations.

## CONCLUSIONS

ED consumption among Erciyes University students was quite widespread. This study determined some characteristics of the students who are at more risk to consume ED. Students need urgent education and awareness programs, especially about the EDs’ potential hazard effects. Legal regulations regarding production, marketing and advertising of EDs’ must be observed.

### Author’s Contribution

**AB:** Planned the study, prepared and finally approved the manuscript.

**BO:** Collected and entered the data, drafted and finally approved the manuscript.

**OG:** Selected the topic, planned the study, analyzed the data, edited and finally approved the manuscript.
